# Prevalence and Co-occurrence of Alcohol, Nicotine, and Other Substance Use Disorder Diagnoses Among US Transgender and Cisgender Adults

**DOI:** 10.1001/jamanetworkopen.2020.36512

**Published:** 2021-02-04

**Authors:** Jaclyn M. W. Hughto, Emily K. Quinn, Michael S. Dunbar, Adam J. Rose, Theresa I. Shireman, Guneet K. Jasuja

**Affiliations:** 1 Department of Behavioral and Social Sciences, Brown University School of Public Health, Providence, Rhode Island; 2 Center for Health Promotion and Health Equity, Brown School of Public Health, Brown University, Providence, Rhode Island; 3 Department of Epidemiology, Brown University School of Public Health, Providence, Rhode Island; 4 Biostatistics and Epidemiology Data Analytics Center, Boston University School of Public Health, Boston, Massachusetts; 5 RAND Corporation, Pittsburgh, Pennsylvania; 6 School of Public Health, Hebrew University, Jerusalem, Israel; 7 Department of Health Services, Policy, and Practice, Brown University School of Public Health, Providence, Rhode Island; 8 Center for Gerontology and Healthcare Research, Brown University, Providence, Rhode Island; 9 Center for Healthcare Organization and Implementation Research, Edith Nourse Rogers Memorial Veterans Affairs Medical Center, Bedford, Massachusetts; 10 Department of Health Law, Policy, and Management, Boston University School of Public Health, Boston, Massachusetts; 11 OptumLabs, Eden Prairie, Minnesota

## Abstract

**Importance:**

Substance use disorders are a major source of morbidity and mortality in the United States. National data comparing the prevalence of substance use disorder diagnoses (SUDDs) among transgender and cisgender individuals are lacking in the United States.

**Objectives:**

To investigate the prevalence of SUDDs among transgender and cisgender adults and to identify within-group and between-group differences by age, gender, and geographic location.

**Design, Setting, and Participants:**

This cross-sectional study used the OptumLabs Data Warehouse to analyze deidentified claims from approximately 74 million adults aged 18 years or older enrolled in commercial or Medicare Advantage insurance plans in 2017. A total of 15 637 transgender adults were identified based on a previously developed algorithm using a combination of *International Classification of Diseases, Tenth Revision *(*ICD-10*) transgender-related diagnosis and procedure codes and sex-discordant hormone prescriptions. A cohort of 46 911 cisgender adults was matched to the transgender cohort in a 3:1 ratio based on age and geographic location.

**Main Outcomes and Measures:**

SUDDs, based on *ICD-10* codes, were assessed overall and compared between transgender and cisgender cohorts and by geographic region (ie, Northeast, Midwest, South, and West); age groups (eg, 18-25, 26-30, 31-35 years), and gender (ie, transfeminine [TF; assigned male sex at birth, identify along feminine gender spectrum], transmasculine [TM; assigned female sex at birth, identify along masculine gender spectrum], male, and female).

**Results:**

In this study of 15 637 transgender adults (4955 [31.7%] TM) and 46 911 cisgender adults (23 664 [50.4%] men), most (8627 transgender adults [55.2%]; 51 762 cisgender adults [55.2%]) were aged between 18 and 40 years, and 6482 transgender adults (41.5%) and 19 446 cisgender adults (41.5%) lived in the South. Comparing transgender to cisgender groups, significant differences were found in the prevalence of a nicotine (2594 [16.6%] vs 2551 [5.4%]; *P* < .001), alcohol (401 [2.6%] vs 438 [0.9%]; *P* < .001), and drug (678 [4.3%] vs 549 [1.2%]; *P* < .001) SUDDs. Among transgender adults, cannabis was the most prevalent drug SUDD (321 [2.1%]), followed by opioid SUDD (205 [1.3%]) and cocaine SUDD (81 [0.5%]), whereas among cisgender adults, cannabis and opioid SUDDs were equally prevalent (cannabis, 186 [0.4%]; opioid, 207 [0.4%]), followed by cocaine SUDD (59 [0.1%]).

**Conclusions and Relevance:**

In this study, the prevalence of SUDDs was significantly elevated among transgender adults relative to their cisgender peers. These findings underscore the need for culturally tailored clinical interventions to treat substance use disorder in transgender populations.

## Key Points

**Question:**

What is the prevalence of substance use disorder diagnoses (SUDDs) among US transgender and cisgender adults?

**Findings:**

In this study of 15 637 transgender and 46 911 cisgender adults, transgender adults had a significantly higher prevalence of nicotine, alcohol, and drug SUDDs than cisgender adults.

**Meaning:**

In this study, transgender adults experienced elevated levels of SUDD relative to their cisgender peers, suggesting that effective, culturally tailored SUDD treatment interventions are warranted.

## Teaser

This cross-sectional study investigates the prevalence of SUDDs among transgender and cisgender adults and identifies within-group and between-group differences by age, gender, and geographic location.

## Introduction

Transgender people, whose gender identity differs from their assigned birth sex and who may access hormones or surgery to align their physical gender expression with their gender identity, are at risk of substance abuse and dependence.^1,2,3,4,5,6,7,8^ Research suggests that substance misuse and related disorders are in part associated with some transgender people’s reliance on substances to cope with the psychological toll of discrimination.^9,10,11^ While research has captured the burden of substance misuse among transgender people,^1,2,3,4,5,6,7^ estimates of substance use disorder (SUD) are limited and vary considerably (ie, 3.9% to 47.2%) depending on the sample and SUD type.^3,5,12,13,14,15,16,17,18^

Much of the research documenting the burden of substance use and related disorders among transgender individuals has relied on geographically limited, survey-based research that often focuses on high-risk subgroups, including younger transgender people and transfeminine (TF) people.^2,3,4,19,20,21^ In addition to the frequent reliance on self-reported outcomes, survey-based research only collects data from individuals who self-identify as transgender and elect to participate in research, which raises concerns about the generalizability of findings. Conversely, health care administrative databases enable the identification of large, geographically representative cohorts of transgender individuals and allow for comparisons to be made between cisgender (ie, nontransgender) and transgender people. Despite the benefits of using administrative claims databases to study substance use disorder diagnoses (SUDDs) among transgender people, few studies use this approach. Furthermore, those that do typically focus on narrow populations (eg, transgender veterans),^17,18^ fail to report the full range of SUDDs (eg, nicotine, alcohol, cannabis, opioid, cocaine),^12,13,16,17,18^ or fail to examine differences by gendered subgroups (eg, TF vs transmasculine [TM] people)^12,13,17,18^ or across geographic regions.^12,13,16,17,18^

The present study aimed to fill these gaps by using a large, national administrative claims database to identify the distribution of SUDDs among transgender and cisgender adults across key subgroups defined by age, gender, and geographic region. The analyses were driven by the overarching goal of identifying subpopulations at greatest risk of SUDDs so that culturally tailored clinical interventions can be developed to treat SUDDs among the most at-risk segments of the transgender population.

## Methods

We conducted a cross-sectional analysis of the OptumLabs Data Warehouse (OLDW), which includes deidentified claims data for commercially insured and Medicare Advantage enrollees. The patient-level information in the OLDW comprises enrollment, medical claims, and pharmacy claims across care settings. Our study sample was drawn from approximately 74 million adults (ie, ≥18 years of age) enrolled in commercial or Medicare Advantage plans in 2017. The study was approved by the institutional review boards of Boston University and the RAND Corporation. Given that this is a secondary data analysis of a deidentified insurance claims dataset, collection of written informed consent was neither possible nor required. This study is reported in accordance with the Strengthening the Reporting of Observational Studies in Epidemiology (STROBE) reporting guideline.

### Cohort Identification and Stratification

To minimize missing data, only individuals who were enrolled for all of 2017 and had at least 5 medical claims reported in 2017 were included in this analysis. Using our previously developed algorithm,^22^ we identified a cohort of transgender adults using a combination of *International Classification of Diseases, Ninth* (*ICD-9*) and *ICD-10* diagnostic codes specific to transgender individuals; *Common Procedural Terminology* codes for transgender-related surgical procedures; and the use of sex-discordant hormones. A cisgender cohort was matched 3:1 to the transgender cohort based on birth year and geographic region (ie, Northeast, South, Midwest, West).

### Measures

#### Outcomes

SUDDs were identified using *ICD-10* codes from health care encounter claims in 2017 and included alcohol, nicotine, cannabis, cocaine, and opioids (see eAppendix in the Supplement). The *ICD-10* also includes codes for other psychoactive substance-related diagnoses. Due to the low prevalence of sedative, stimulant, and hallucinogen SUDDs, individuals with diagnosis codes related to these disorders were included in the other SUDD group. To compare findings with the US general population,^23^ nonalcohol and nonnicotine SUDDs were combined to create an indicator of drug SUDDs. The polysubstance SUDD variable included individuals with 2 or more SUDDs.

#### Demographic Characteristics

Age was divided into 9 categories, as follows: 18 to 25; 26 to 30; 31 to 35; 36 to 40; 41 to 45; 45 to 50; 51 to 55; 56 to 60; and 61 years or older. For cisgender people, gender was categorized as male vs female. For transgender people, gender was categorized as TF, TM, and unknown.^22^ TF individuals were defined as transgender people who received feminizing hormones and/or had received a feminizing surgical procedure (eg, vaginoplasty). TM individuals were defined as transgender people who received masculinizing hormones and/or had sentinel surgeries (eg, metoidioplasty or phalloplasty). The remaining transgender cohort, most of whom were classified as transgender based on a gender-related diagnostic code (eg, gender identity disorder), did not have claims for hormones or surgeries that allowed for their categorization as TF or TM.

### Statistical Analysis

Analyses were performed using SAS version 9.4 (SAS Institute). Due to our cohort inclusion criteria (ie, full year of enrollment, minimum of 5 claims), there were no missing data. Period prevalence estimates for each SUDD were calculated for 2017 among transgender and cisgender individuals. We used 2-tailed χ^2^ tests, with statistical significance set at *P* < .05, to assess within-group differences (among transgender people and among cisgender people) and between-group differences (across transgender and cisgender people) in SUDD by geographic region, age, and gender subgroup (TF vs TM; male vs female). Transgender individuals with an unknown gender were excluded from the within-group, gender-stratified analysis.

## Results

In this US study of 15 637 transgender individuals (2079 [13.3%] TF; 4955 [31.7%] TM; 8603 [55.0%] unknown gender) and 46 911 cisgender individuals (23 664 [50.4%] male; 23 247 [49.6%] female), most (8627 transgender adults [55.2%]; 51 762 cisgender adults [55.2%]) were between 18 and 40 years of age, and the largest proportion lived in the South (6482 transgender adults [41.5%]; 19 446 cisgender adults [41.5%]) (Table 1). Significantly more transgender people than cisgender people had a nicotine (2594 [16.6%] vs 2551 [5.4%]; *P* < .001), alcohol (401 [2.6%] vs 438 [0.9%]; *P* < .001), or drug (678 [4.3%] vs 549 [1.2%]; *P* < .001) SUDD. Significantly more transgender individuals had a polysubstance SUDD than cisgender individuals (310 [2.0%] vs 245 [0.5%]; *P* < .001). Among transgender individuals, cannabis was the most prevalent drug SUDD (321 [2.1%]), followed by opioid (205 [1.3%]) and cocaine (81 [0.5%]) SUDDs. Among cisgender people, cannabis and opioid SUDDs were equally prevalent (cannabis, 186 [0.4%]; opioid, 207 [0.4%]), followed by cocaine SUDD (59 [0.1%]).

**Table 1.** Characteristics of a Cohort of 15 637 Transgender Adults and a Cohort of 46 911 Cisgender Adults in the US, 2017

Characteristic	No. (%)		Bivariate comparisons^a^	
	Transgender (n = 15 637)	Cisgender (n = 46 911)	χ^2^	*P* value
Geographic region^b^				
Northeast	1963 (12.6)	5889 (12.6)	NA	NA
Midwest	3760 (24.0)	11 280 (24.0)		
West	3432 (21.9)	10 296 (21.9)		
South	6482 (41.5)	19 446 (41.5)		
Age, y				
18-25	4084 (26.1)	12 252 (26.1)	NA	NA
26-30	1858 (11.9)	5574 (11.9)		
31-35	1369 (8.8)	4107 (8.8)		
36-40	1316 (8.4)	3948 (8.4)		
41-45	1167 (7.5)	3501 (7.5)		
45-50	1302 (8.3)	3906 (8.3)		
51-55	1257 (8.0)	3771 (8.0)		
56-60	1154 (7.4)	3462 (7.4)		
≥61	2130 (13.6)	6390 (13.6)		
Gender spectrum				
Transfeminine	2079 (13.3)	NA	NA	NA
Transmasculine	4955 (31.7)	NA		
Unknown gender	8603 (55.0)	NA		
Male	NA	23 664 (50.4)		
Female	NA	23 247 (49.6)		
SUDDs				
Polysubstance	310 (2.0)	245 (0.5)	284.3	<.001
Nicotine	2594 (16.6)	2551 (5.4)	1931.7	<.001
Alcohol and drug	887 (5.7)	820 (1.7)	680.4	<.001
Alcohol	401 (2.6)	438 (0.9)	235.7	<.001
Drug	678 (4.3)	549 (1.2)	611.1	<.001
Cannabis	321 (2.1)	186 (0.4)	400.2	<.001
Cocaine	81 (0.5)	59 (0.1)	80.8	<.001
Opioid	205 (1.3)	207 (0.4)	135.6	<.001
Another drug^c^	265 (1.7)	183 (0.4)	280.7	<.001

Abbreviations: NA, not applicable; SUDD, substance use disorder diagnoses.

^a^ Bivariate comparisons are not reported for age and geographic region because the cisgender cohort was matched 3 to 1 to the transgender cohort based on these characteristics.

^b^ Hawaii and Alaska are included in the West according to the US Census. US territories (eg, Puerto Rico and Guam) are not included in US Census regions; thus the approximately 100 individuals without an assigned census region were included in the South.

^c^ Another drug SUDD includes hallucinogens, sedatives, stimulants, and the diagnosis of other SUD not otherwise specified.

When examining differences in SUDD by geographic region (Table 2), transgender people within each region had a significantly higher prevalence of all SUDDs relative to cisgender people, except for cocaine SUDD in the West. When comparing the prevalence of SUDDs among transgender people, those in the Northeast had a significantly higher prevalence of nicotine (352 [17.9%]), alcohol (65 [3.3%]), cannabis (62 [3.2%]), cocaine (19 [1.0%]), opioid (32 [1.6%]), and another (49 [2.5%]) SUDD compared with transgender people in other regions. A similar pattern was observed among cisgender people; with the exception of nicotine SUDD, which was highest among cisgender people in the Midwest (695 [6.2%]) followed by those in the South (1165 [6.0%]), Northeast (281 [4.8%]), and West (410 [4.0%]) (*P* < .001).

**Table 2.** Prevalence of SUDDs by Geographic Region Among 15 637 Transgender and 56 911 Cisgender Adults, 2017^a^

SUDD	No. (%)		Bivariate comparisons^b^	
	Transgender (n = 15 637)	Cisgender (n = 46 911)	χ^2^	*P* value
**Northeast**				
Total adults, No.	1963	5889		
Nicotine	352 (17.9)	281 (4.8)	344.0	<.001
Alcohol	65 (3.3)	72 (1.2)	37.5	<.001
Cannabis	62 (3.2)	33 (0.6)	83.1	<.001
Cocaine	19 (1.0)	12 (0.2)	21.9	<.001
Opioid	32 (1.6)	32 (0.5)	21.5	<.001
Another drug^c^	49 (2.5)	36 (0.6)	48.9	<.001
**South**				
Total adults, No.	6842	19 446		
Nicotine	1103 (17.0)	1165 (6.0)	740.4	<.001
Alcohol	146 (2.3)	157 (0.8)	87.9	<.001
Cannabis	103 (1.6)	59 (0.3)	129.4	<.001
Cocaine^d^	>24 (>0.4)	>24 (>0.1)	NA	<.001
Opioid	89 (1.4)	92 (0.5)	56.8	<.001
Another drug^c^	110 (1.7)	79 (0.4)	111.9	<.001
**Midwest**				
Total adults, No.	3760	11 280		
Nicotine	673 (17.9)	695 (6.2)	469.9	<.001
Alcohol	118 (3.10)	112 (1.0)	86.2	<.001
Cannabis	94 (2.5)	49 (0.4)	127.8	<.001
Cocaine	27 (0.7)	12 (0.1)	40.8	<.001
Opioid	51 (1.4)	42 (0.4)	44.4	<.001
Another drug^c^	58 (1.5)	35 (0.3)	69.7	<.001
**West**				
Total adults, No.	3432	10 296		
Nicotine	466 (0.1)	410 (4.0)	396.8	<.001
Alcohol	72 (0.2)	97 (0.9)	28.3	<.001
Cannabis	62 (0.2)	45 (0.4)	62.4	<.001
Cocaine^d^	<11 (<0.3)	<11 (<0.1)	NA	.01
Opioid	33 (0.1)	41 (0.4)	15.2	<.001
Another drug^c^	48 (0.1)	33 (0.3)	51.0	<.001

Abbreviation: SUDD, substance use disorder diagnosis.

^a^ Hawaii and Alaska are included in the West according to the US Census. US territories (eg, Puerto Rico and Guam) are not included in US Census regions; thus the approximately 100 individuals without an assigned census region were included in the South.

^b^ Bivariate comparisons are between transgender and cisgender people in each region of the United States. Comparisons were also made in the frequency of each SUDD among transgender individuals across regions and among cisgender people across regions. Among transgender people, significant within group differences (*P* < .05) were observed for all SUDDs across regions with the exception of cocaine (*P* = .18). Among cisgender people, significant within group differences (*P* < .05) were observed for all SUDDs across regions with the exception of cocaine (*P* = .21) and opioid (*P* = .31) SUDDs.

^c^ Another drug SUDD includes hallucinogens, sedatives, stimulants, and the diagnosis of other SUD not otherwise specified.

^d^ Exact values are not reported to prevent back calculation and protect the confidentiality of individuals.

The Figure presents differences in the prevalence of SUDDs between transgender and cisgender people across age groups. Transgender people in each age group had a significantly higher prevalence of polysubstance, alcohol, nicotine, and drug SUDD relative to cisgender people. For transgender and cisgender people, polysubstance (transgender, 134 of 4084 [3.3%]; cisgender, 122 of 12 252 [1.0%]), alcohol (transgender, 130 [3.2%]; cisgender, 156 [1.3%]), and drug (transgender, 269 [6.6%]; cisgender, 243 [2.0%]) SUDDs were highest among those aged 18 to 25 years. The inverse was observed with regard to nicotine SUDD, which was highest among those aged 61 years or older (transgender, 575 of 2130 [27.0%]; cisgender, 700 of 6390 [11.0%]).

**Figure.**
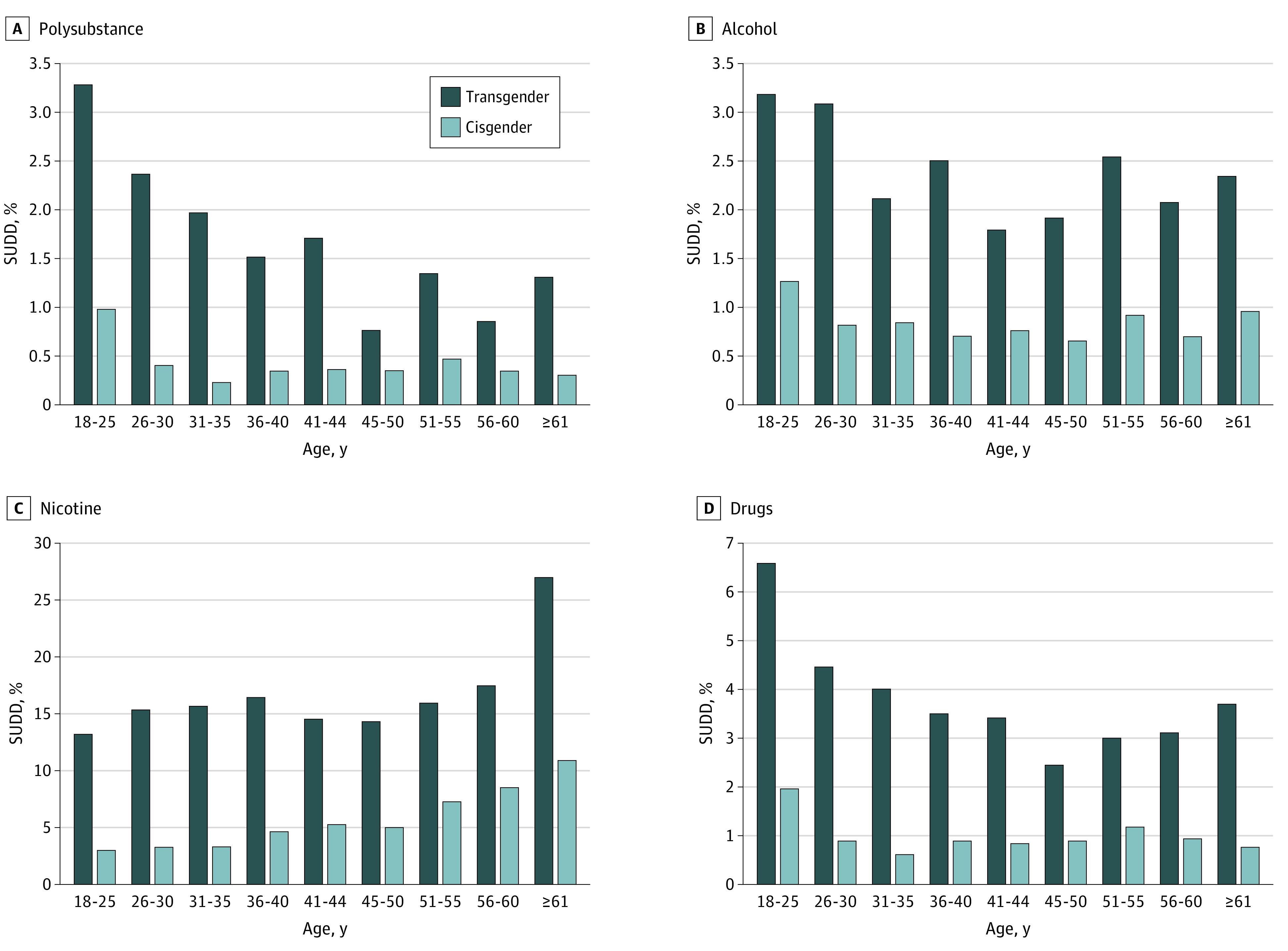
Frequency of Substance Use Disorder Diagnoses (SUDDs) by Age Among US Transgender (n = 15 637) and Cisgender (n = 46 911) Adults, 2017 Drug SUDD includes cannabis, cocaine, opioids, and other drugs, including stimulants, hallucinogens, and sedatives.

Table 3 presents stratified analyses between the cisgender sample and a subset (7034 [45.0%]) of the transgender sample for which gender spectrum (TF or TM) could be determined. Among the transgender subsample, TF people had a significantly higher prevalence of every SUDD relative to TM people, with TF individuals having approximately 3 times the prevalence of polysubstance (49 of 2079 [2.4%] vs 39 of 4955 [0.8%]; *P* < .001), cannabis (48 [2.3%] vs 42 [0.8%]; *P* < .001), and cocaine (18 [0.9%] vs <0.3%; *P* < .001) SUDDs, and 2.3 times the prevalence of alcohol SUDD (53 [2.5%] vs 56 [1.1%]; *P* < .001). Among cisgender people, male adults compared with female adults had approximately 2 times the prevalence of polysubstance (159 of 23 247 [0.7%] vs 87 of 23 664 [0.4%]; *P* < .001), alcohol (283 [1.2%] vs 155 [0.7%]; *P* < .001), cannabis (115 [0.5%] vs 72 [0.3%]; *P* < .001), and cocaine (35 [0.2%] vs 24 [0.1%]; *P* < .001) SUDDs.

**Table 3.** Substance Use Disorder Diagnoses by Gender Among Transfeminine Individuals, Transmasculine Individuals, Cisgender Male Individuals, and Cisgender Female Individuals in the US, 2017

SUDD	Transgender, No. (%)		Bivariate comparison 1^a^		Cisgender, No. (%)		Bivariate comparison 2^a^	
	Transfeminine (n = 2079)	Transmasculine (n = 4955)	χ^2^	*P* value	Male (n = 23 247)	Female (n = 23 664)	χ^2^	*P* value
Polysubstance	49 (2.4)	39 (0.8)	29.2	<.001	159 (0.7)	87 (0.4)	22.5	<.001
Nicotine	335 (16.1)	551 (11.1)	33.2	<.001	1272 (5.5)	1279 (5.4)	0.1	.75
Alcohol and drug	114 (5.5)	161 (3.2)	19.5	<.001	476 (2)	344 (1.5)	24.1	<.001
Alcohol	53 (2.5)	56 (1.1)	19.3	<.001	283 (1.2)	155 (0.7)	40.1	<.001
Drug	91 (4.4)	123 (2.5)	17.8	<.001	308 (1.3)	241 (1)	9.5	.002
Cannabis	48 (2.3)	42 (0.8)	24.8	<.001	114 (0.5)	72 (0.3)	10.3	.001
Cocaine	18 (0.9)	<11 (0.3)^b^	>14.8	<.001	35 (0.2)	24 (0.1)	2.3	.13
Opioid	23 (1.1)	51 (1.0)	0.1	.77	109 (0.5)	98 (0.4)	0.8	.37
Another drug^c^	31 (1.5)	40 (0.8)	6.9	.01	105 (0.45)	78 (0.3)	4.5	.03

Abbreviation: SUDD, substance use disorder diagnoses.

^a^ Using χ^2^ analyses, bivariate comparisons compare within-group differences in each SUDD category between transfeminine and transmasculine individuals (1) and cisgender male and female individuals (2).

^b^ To protect the confidentiality of individuals, exact values are not reported.

^c^ Another drug SUDD includes hallucinogens, sedatives, stimulants, and the diagnosis of other SUD not otherwise specified.

## Discussion

To our knowledge, this study represents the largest national study of SUDD disparities among transgender and cisgender adults to date. In 2017, the prevalence of polysubstance SUDD among transgender people in our sample was 4 times that of their cisgender peers. Moreover, the prevalence of any drug SUDD among transgender people was 3.6 times that of cisgender people in our sample and approximately 1.6 times that of the US general population in 2017.^23^ Findings underscore the need for future research to examine the SUDD treatment experiences of transgender individuals so that effective clinical interventions can be developed to reduce the burden of SUDD among this population.

When examining SUDDs separately, significant disparities were observed. Notably, nicotine SUDD was the most prevalent SUDD for both transgender and cisgender individuals, although the prevalence was significantly higher among transgender people (16.6%) than cisgender individuals (5.4%) in the sample. While national data comparing the prevalence of diagnosed nicotine disorder among transgender and cisgender people are lacking, prior research among lesbian, gay, bisexual, and transgender (LGBT) populations has documented elevated rates of nicotine use and the need for interventions to mitigate the harms of nicotine consumption among these populations, particularly cigarette smoking.^24,25,26,27^ Regarding cannabis, transgender individuals in this sample had more than 5 times the prevalence of cannabis SUDD than cisgender people and 1.5 times that of the 2017 US general population.^23^ Similarly, transgender individuals in this sample had 4 times the prevalence of cocaine SUDD and 3 times the prevalence of opioid SUDD relative to cisgender people and 1.4 and 1.7 times the prevalence of cocaine and opioid SUDD, respectively, relative to the 2017 US general population.^23^ Research has found that transgender individuals frequently use substances to cope with discrimination.^9,10,11^ While the use of substances such as nicotine, cannabis, cocaine, and opioids may be an effective short-term strategy to cope with the stress of discrimination, these substances carry a myriad of health risks.^28,29,30^ Transgender individuals who use substances to cope with discrimination may benefit from engagement in clinical interventions that promote positive coping strategies such as meditation, exercise, or peer support.^10,31^

Much of the prior substance use research with transgender people is geographically limited, and the national research that does exist routinely fails to explore regional differences in SUDD. In this study, we found that transgender individuals in the Northeast, South, Midwest, and West consistently had higher rates of nicotine, alcohol, and all other SUDDs relative to cisgender individuals within the same region. We also found that transgender people in the Northeast had significantly higher prevalence of nicotine, alcohol, cannabis, cocaine, opioid, and other SUDDs relative to transgender individuals in other regions. The prevalence of alcohol and cocaine SUDD was similarly elevated among cisgender individuals in the Northeast, relative to those in other regions. National 2017 data on US residents aged 12 years and older found that those living in the West had the highest prevalence of past-year drug misuse relative to those in other regions; however, the prevalence of substance use treatment was highest in the Northeast, relative to other regions.^32^ Since health care engagement is required to receive an SUDD, it may be that cisgender and transgender people from the West misuse drugs more, but those in the Northeast have greater access and/or motivation to engage in treatment. Differences in the regional distribution of SUDDs in our study relative to the distribution of SUDDs among the US general population, aged 12 years and older, may be due to the fact that our study included people with insurance aged 18 years and older and older age and insurance may be particularly protective against having an SUDD for those living in the West vs other US regions. Additional research is needed to understand the mechanisms driving geographic disparities in SUDD among cisgender and transgender people in the United States.

When examining differences according to age, our study found that transgender adults aged 18 to 25 years had the highest burden of all SUDDs (except nicotine) relative to older transgender people and cisgender people of all ages. Our study extends findings from much smaller observational studies documenting the high prevalence of disordered alcohol and drug use behaviors among younger transgender people^2,3,5,20,33^ and also aligns with 2017 survey data showing an elevated prevalence of alcohol and drug SUDDs among young adults (aged 18-25 years) relative to older members of the US general population.^23^ Notably, however, the prevalence of nicotine SUDD in the present study doubled among transgender individuals between the ages of 18 and 25 years and those aged 61 years and older and more than tripled among cisgender individuals across age groups. Although US general population data suggest greater nicotine use and dependence among younger adults,^34,35^ concerns about the health risks of smoking and the desire to quit have been shown to increase with age,^36^ and a significantly higher proportion of adults aged 65 years and older report being former smokers than those younger than 65 years.^35,37^ Thus, the age-related disparities in nicotine SUDD documented among transgender and cisgender adults in our sample may reflect a greater recognition of problematic use and elevated treatment-seeking among adults in the older age groups relative to those in the younger age groups. Taken together, these findings highlight the need to detect and treat nicotine SUDD among US adults as they age and underscore the need for early prevention efforts to alleviate the burden of alcohol, cocaine, opioid, and other SUDDs among young adults, particularly those who are transgender.

Extending prior substance use research,^4,21^ our gender-stratified subanalysis of the 7034 transgender people who had received gender-affirming hormones or surgery found that TF individuals had a higher prevalence of nearly every SUDD relative to TM individuals. Survey research has consistently documented the high prevalence of substance use and SUD among TF individuals alone^3,20,38^ and relative to TM people^4,21,39^; however, no national study, to our knowledge, has documented disparities in diagnosed SUD among TF and TM adults until now. The higher prevalence of SUDDs among TF people relative to TM individuals in our study could be attributed to the possibility that TF people experience greater stressors, have fewer adaptive stress management strategies, or clinically present with SUD symptoms at higher rates than TM people.^4,40^ Additionally, consistent with prior research,^16,32,41^ cisgender male adults in our sample also had a higher prevalence of alcohol and drug SUDDs than cisgender female adults; thus, there may be sex-related and developmental factors that lead individuals assigned a male birth sex to engage in heavier substance use and be diagnosed with an SUD more readily than individuals assigned a female birth sex.^41^ Future research is needed to explore the potential factors associated with the differential prevalence of SUDDs across gender subgroups.

Our research underscores the necessity of ensuring access to SUD treatment for transgender people. Findings also support the necessity of tailoring clinical interventions to the highest risk groups including TF people and young adults. Given early research suggesting that many substance use treatment facilities may be ill-equipped to meet the treatment needs of transgender adults with SUDDs^42,43^ and the paucity of evidence-based SUD interventions for this population,^44^ research that examines the treatment experiences and unmet treatment needs of transgender individuals with SUDDs is needed to inform future interventions for this population.

### Limitations

Our analysis has several limitations. We used our previously developed algorithm^22^ that relied on diagnosis and procedure codes to identify transgender individuals with commercial or Medicare Advantage insurance. Moreover, only diagnosed SUDs were assessed. Given that lack of insurance is a barrier to accessing care and receiving an SUDD^45^ and transgender individuals are more likely than the US general population to be uninsured^6^ and face numerous stigma-related barriers to health care engagement,^6,10^ the SUDDs documented here are likely underestimates of the true burden of SUDD in the US transgender population. Additionally, the OLDW does not include self-reported beneficiary-level data on race; therefore, we were unable to explore racial differences in SUDDs. Given that greater substance use had been documented among transgender people of color than white transgender people,^3,46^ future population-level research should aim to explore within-group SUDD disparities by race.

Additionally, our algorithm^22^ could only identify transgender individuals with a transgender-related diagnosis or gender-affirming hormones or surgery. Furthermore, our necessary reliance on feminizing and masculinizing hormones and procedures to categorize transgender individuals as TF and TM meant that the gender of 55% of the transgender cohort could not be categorized and these individuals were subsequently excluded from the gender-stratified subanalysis. Thus, the gender-stratified, transgender subanalysis is only representative of transgender individuals who have accessed gender-affirming hormones or surgery. Notably, across community-based studies, nonbinary individuals have comprised 35% to 42% of transgender samples^6,47,48^ and many nonbinary individuals do not use gender-affirming hormones or procedures^6,49,50^; thus, it is likely that most individuals who were excluded from our gender-stratified analyses were nonbinary people who had not accessed hormones or surgery. Furthermore, research finds that TF people are less likely to receive various forms of gender-affirming services than TM people^16,51^ and nonreceipt of gender-affirming care is associated with substance use.^4,52^ Thus, it is likely that the burden of SUDD would have been higher among both the TF and TM subsamples if individuals who had not accessed gender-affirming care had been included, with potentially wider SUDD disparities observed between the TF and TM subsamples. National data are needed to characterize SUDD disparities among TF, TM, and nonbinary people who have and have not accessed gender-affirming medical care.

## Conclusions

To our knowledge, this is the largest national study to document within-group and between-group disparities in SUDDs among US transgender and cisgender adults. Transgender individuals in our study had significantly higher rates of SUDDs compared with cisgender individuals, a pattern that persisted when transgender and cisgender cohorts were compared across age groups and geographic areas. These findings highlight the need to ensure access to high-quality SUD treatment for transgender individuals as well as additional research to understand facilitators and barriers to SUD treatment engagement for this population. Such research can inform the development of novel public health interventions to prevent and treat SUD among transgender people in the United States.

## Notes

Supplement. **eAppendix. **List of Substance Use Disorder Diagnoses Codes
