# Errors in Abstract, Methods, Results, Figure, and Tables

**DOI:** 10.1001/jamanetworkopen.2021.3314

**Published:** 2021-03-02

In the Original Investigation titled “Prevalence and Co-occurrence of Alcohol, Nicotine, and Other Substance Use Disorder Diagnoses Among US Transgender and Cisgender Adults,”^1^ published February 4, 2021, there were errors in the Abstract, Methods, Results, the Figure, Table 1, and Table 2. In the Abstract, Results, and Table 1, the number of male cisgender adults should have read 23 247 and the number of cisgender female adults should have read 23 664. In the Methods, Table 1, and the Figure, the age ranges should have read 41 to 45 years and 46 to 50 years. In the results, the *P *values for the comparison of cannabis and cocaine substance use disorder diagnoses were incorrect. The *P *value for cannabis should have been .001; for cocaine, .13. The title of Table 2 should have indicated that there were 49 611 cisgender adults in the sample. This article has been corrected.^1^
