# Supplementary material for: Prevalence and Co-occurrence of Alcohol, Nicotine, and Other Substance Use Disorder Diagnoses Among US Transgender and Cisgender Adults
Source: JAMA Netw Open. 2021 Feb 4;4(2):e2036512. doi: 10.1001/jamanetworkopen.2020.36512 (PMC7862992; doi:10.1001/jamanetworkopen.2020.36512)
Supplement: Supplement. — eAppendix. List of Substance Use Disorder Diagnoses Codes [file jamanetwopen-e2036512-s001.pdf]

Supplemental Online Content

Hughto JMW, Quinn EK, Dunbar MS, Rose AJ, Shireman TI, Jasuja GK. Prevalence and co-occurrence of alcohol, nicotine, and other substance use disorder diagnoses among US transgender and cisgender adults. *JAMA Netw Open*. 2021;4(2):e2036512. doi:10.1001/jamanetworkopen.2020.36512

eAppendix. List of Substance Use Disorder Diagnoses Codes

This supplemental material has been provided by the authors to give readers additional information about their work.

© 2021 Hughto JMW et al. *JAMA Network Open.*

eAppendix. List of Substance Use Disorder Diagnoses Codes

<u>Alcohol:</u>

ICD10 F10.x G62.1 I42.6 K29.2 K70.x T51.0

<u>Cannabis</u>

ICD10 F12.x

<u>Opioid</u>

ICD10 F11.x

<u>Sedatives</u>

ICD10 F13.x

<u>Cocaine</u>

ICD10 F13.x

<u>Nicotine</u>

ICD10 F17.x Z87.8

<u>Other Drugs (includive of stimulant and hallucinogen SUDD)</u>

ICD10 F19.x F18.x F15.x F16.x

<u>Polysubstance*</u>

Two or more of any of the above codes in a give year

© 2021 Hughto JMW et al. *JAMA Network Open.*
